# Role of Alternative Lipid Excipients in the Design of Self-Nanoemulsifying Formulations for Fenofibrate: Characterization, *in vitro* Dispersion, Digestion and *ex vivo* Gut Permeation Studies

**DOI:** 10.3389/fphar.2018.01219

**Published:** 2018-11-06

**Authors:** Aws Alshamsan, Mohsin Kazi, Mohamed M. Badran, Fars Kaed Alanazi

**Affiliations:** ^1^Department of Pharmaceutics, College of Pharmacy, King Saud University, Riyadh, Saudi Arabia; ^2^Nanobiotechnology Unit, College of Pharmacy, King Saud University, Riyadh, Saudi Arabia

**Keywords:** lipid-based formulation, self-nanoemulsifying drug delivery systems (SNEDDS), *in vitro* dynamic dispersion, *in vitro* digestion, fenofibrate, solubility improvement

## Abstract

**Background:** The choice of lipid excipients and their origin are crucial determinant factors in the design of self-nanoemulsifying drug delivery system (SNEDDS).

**Aim:** To investigate the aspects of alternative excipients which can influence the development of efficient SNEDDS and determine the fate of fenofibrate in aqueous media.

**Methods:** SNEDDS of two groups (a and b) were developed using Cremercoor MCT/Capmul MCM and Kollisolv MCT/Imwitor 742 blended oils and water soluble surfactants (to improve lipid polarity) for the model anti-cholesterol drug fenofibrate. Visual assessment was employed and droplet size measurement was taken into initial consideration for optimized SNEDDS. Further SNEDDS optimizations were done on the basis of maximum drug loading by equilibrium solubility studies and maximum solubilized drug upon aqueous dispersion by dynamic dispersion studies. *In vitro* lipolysis was examined under simulated Fed and Fasted conditions. Intestinal permeability study of the optimal SNEDDS formulation was compared with the raw fenofibrate dispersion using non- everted “intestinal sac technique.”

**Results:** Initial characterization and solubility studies showed that mixed glycerides of Kollisolv MCT/Imwitor 742 (group b) containing formulations generated highly efficient SNEDDS as they are stable and produced lower nanodroplets with higher drug loading (group b) as compared to mixed glycerides of Cremercoor MCT/Capmul MCM (group a). *In vitro* dispersion and digestion studies confirmed that SNEDDS of group b (polar mixed glycerides) can retain high amount of drug (99% drug in solution for more than 24 h time) in dispersion media and have high recovery after digestion. The results from the permeability assessment confirmed that fenofibrate had 4.3-fold increase with F3b SNEDDS compared with the control.

**Conclusion:** SNEDDS formulations containing alternative excipients (Kollisolv MCT/Imwitor 742 blend) could be a potential oral pharmaceutical product in taking anti-hyperlipidaemic agent fenofibrate to the systemic circulation as solubilized form.

## Introduction

**GRAPHICAL ABSTRACT:**
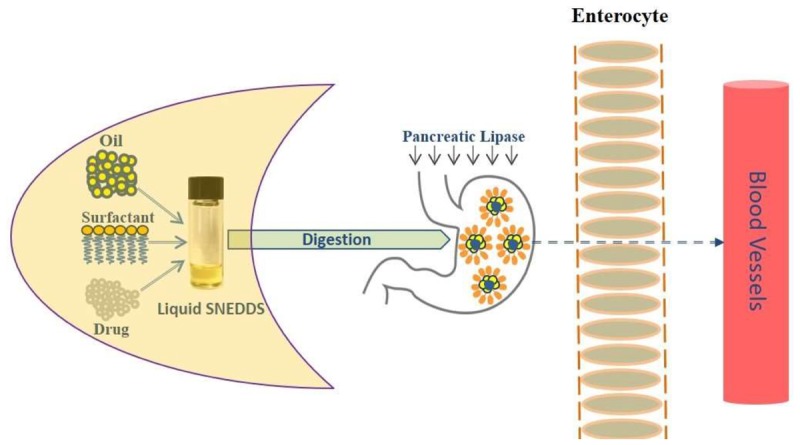
Improving fenofibrate solubility using lipid-based self-nanoemulsifying systems. Drug solubilization required for drug absorption for an anticipated systemic effect of an orally administered drug. Self-nanoemulsifying drug delivery systems (SNEDDS) have generated significant interest across the pharmaceutical industry as potentially innovative systems to maintain poorly water soluble drug in solubilized form for rapid absorption. **Schematic Diagram:** Intestinal drug transport mechanism after ingestion of lipid-based SNEDDS dosage form.

Hyperlipidaemia is defined as elevated lipid concentration in bloodstream. It is manifested clinically as hypercholesterolemia or hypertriglyceridemia. Plasma proteins with which lipids are associated and remain in the dissolved form in blood are called lipoproteins (Najib, 2002; Cho and Park, 2014). There are divided into major classes including chylomicrons, very low-density lipoproteins (VLDL), low-density lipoproteins (LDL), or high-density lipoproteins (HDL). Hyperlipidaemia is the prime risk factor for atherosclerosis and cardiovascular disease which caused approximately 800,000 deaths in 2005 as first killer in United States (Farnier et al., 2007) with yet increasing number of incidents. Approximately, 33.5% of adults in United States and 50% adults in Saudi Arabia have high cholesterol level (Najib, 2002), which possesses a significant threat and can cause considerable deaths in the regions.

World Health Organization (WHO) reported that out of the total global cardiovascular disease (CVD)-associated deaths, heart attacks, i.e., myocardial infarction and strokes were responsible for 42.1 and 35.8% deaths, respectively (Farnier et al., 2007). The INTERHEART and INTERSTROKE studies revealed that dyslipidaemia is one of the most common risk factors for heart attacks and strokes worldwide. CVD was estimated to account almost half of the deaths in Saudi Arabia, 42% respectively.

Most common groups of lipid-lowering drugs are statins and fibrates. Like all fibrates, fenofibrate has been shown effective in reducing the formation and breakdown of cholesterol/triglycerides in the body mainly in peroxisomes and partly in mitochondria by stimulating *β*-oxidation of fatty acids (Miller and Spence, 1998), yet it exhibits low aqueous solubility and poor absorption after oral administration that demands significant delivery strategies.

**FIGURE 1:**
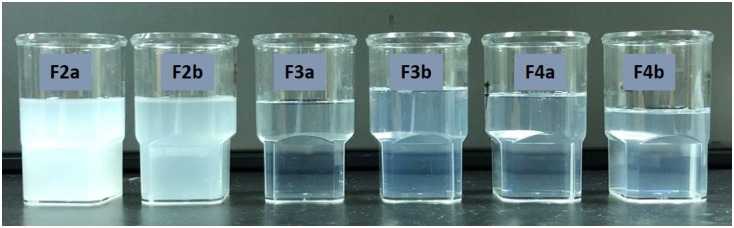
Typical physical appearance of immediately dispersed formulations. The formulation used in the experiment were aqueous dispersions of the “drug free” formulations F2–F4 representing the most efficient self-nanoemulsifying systems (SNEDDS).

Fenofibrate was chosen as the model drug as it has shown previously extensive precipitation in the intestinal media from self-nanoemulsifying drug delivery systems (SNEDDS) containing conventional water soluble lipid excipients during dispersion and digestion. Fenofibrate is classified as class II drug according to Biopharmaceutical Classification System (BCS). It is a prodrug with high dose number converted rapidly to active metabolite fenofibric acid by hydrolysis of ester molecule after oral administration (Ashok and Pradeep, 2007; Alamri et al., 2017). It is poorly soluble in aqueous media with 35% absolute bioavailability. It has chemical, pharmacological, and clinical similarities with other fibrate drugs such as clofibrate and gemfibrozil. As shown in Figure 1, fenofibrate is a non-electrolyte small lipophilic molecule (MW 360.8) with low aqueous solubility (3 μg/mL) and high octanol/water partition coefficient (log P-4.6).

Lipid-based drug delivery system is one of the most popular approaches, which range from simple solutions of drugs in lipids to complex mixtures of oils, surfactants, co-surfactants and co-solvents. One of these mixtures is characterized as SNEDDS (Mohsin et al., 2012; Shahba et al., 2012).

SNEDDS is an emerging formulation approach as nano-carriers during the preparation of pharmaceutical dosage forms for poorly water-soluble drugs and has wider commercial application. Its properties strongly depend on the selected lipids, surfactants, lipid-surfactant mixing ratios and their purity from the manufacturer. In addition, the use of lipid excipients from different manufacturer can provide SNEDDS with different characters due to their variations in polarities. SNEDDS spread readily in the GI tract and take up huge amount of water thus produce transparent self-nanoemulsifying systems with a droplet size between 20 and 200 nm. In comparison to many other drug delivery systems, SNEDDS can maintain drug in solution for considerable amount of time prior to absorption, increase intestinal absorption (Ibrahim et al., 2014) and have the potential to reduce the extent of efflux and pre-systemic metabolism. These effects can enhance the bioavailability and provide the desired reproducible pharmacokinetic profile of orally administered drugs such as fenofibrate (Mohsin et al., 2016).

The aim of this work is to develop optimum fast-dissolving liquid SNEDDS– a suite of products – for today’s most promising water insoluble anti-hyperlipidaemic drug, fenofibrate. Our approach of using alternative excipients in an oral liquid capsule dosage form is expected to increase solubility in the GI contents and consequently improve bioavailability and reduce pill burden. In addition, special attention was given to understand the role of excipients from different manufacturer on SNEDDS development.

## Materials and Methods

### Materials

All chemicals/materials used in the studies were obtained from well-known commercial suppliers and used without further purification. Fenofibrate, (2-[4-(4-chlorobezoyl) phenoxy]-2-methylpropionic acid 1-methylethyl ester) was purchased from Sigma-Aldrich Co, St. Louis, MO, United States. All lipids (oils, non-ionic surfactants), their compositions and suppliers’ details are given in Table 1. HPLC grade methanol and lipolysis buffer contents, i.e., saline solution, sodium dihydrogen phosphate and sodium chloride were purchased from BDH laboratory supplies (BDH Chemicals Ltd., Poole, United Kingdom). Simulated Intestinal Fluid (SIF) powder, was obtained from biorelevant.com (Corydon, Surrey, CR2 0BS, United Kingdom) to make fasted and fed state intestinal fluid (FaSSIF and FeSSIF). Porcine pancreatin (8_USP specifications activity) were from Sigma Chemical Co. St Louis, MO, United States. Digestion inhibitor, 4 bromophenylboronic acid (4-BPBA) was obtained from Aldrich Chemicals Co. St Louis, MO, United States. 1 M sodium hydroxide (Titrisol), was purchased from Merck, Darmstadt, Germany. High purity Milli-Q water was obtained through a Milli-Q pure lab flex (Veolia water).

**Table 1:** Description of the lipids and their chemical structures.

Name	Function and composition	Chemical structure	Supplier
Fenofibrate	Drug, Non ionizable ester compound (log P: 5.24)	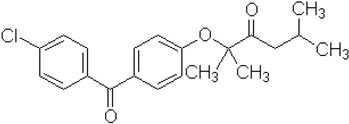	Sigma Aldrich Co, St. Louis, MO, United States
TO-106V	Surfactant, PEG-6 sorbitan oleate water insoluble (HLB = 10)	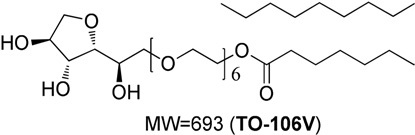	Nikko Chemicals Co., Tokyo, Japan.
HCO-30	Surfactant, PEG-30 hydrogenated castor oil, water-soluble (HLB = 11)	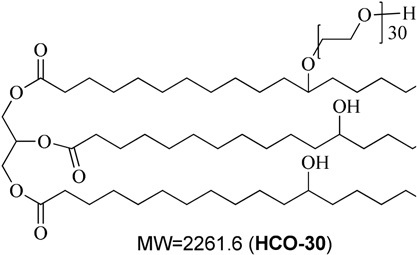	Nikko Chemicals Co., Tokyo, Japan.
Capmul- MCM (C6), Capmul-MCM (C8), Capmul-MCM (C10)	Secondary oil, Caproic 1% C_6_, Caprylic C_8_-85%/Capric C_10_-14% Glycerides Mono-diglycerides (monoglycerides 80%)	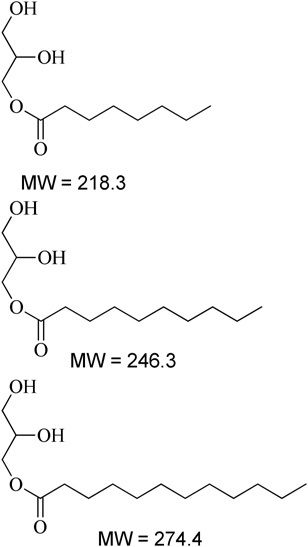	Abitec Corporation, Germany
Imwitor 742 (C6, C8, C10)	Secondary oil (used as alternative excipient), Caprylic C_8_-70%/Capric C_10_-27% Glycerides (monoglycerides 52%, diglycerides 35%, and triglycerides 10%)	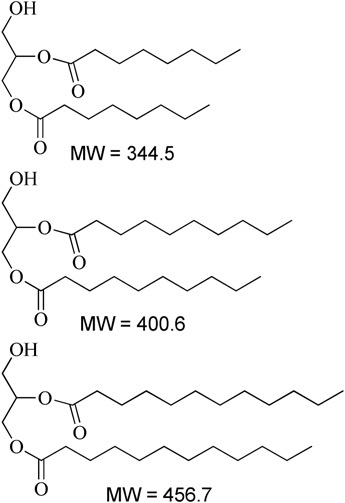	Cremer Oleo GmbH & Co. KG. Hamburg, Germany.
Cremercoor-MCT-70/30 (C6), Cremercoor-MCT-70/30 (C8), Cremercoor-MCT-70/30 (C10),	Primary oil, Caprylic/capric acid triglycerides (2% caproic C_6_, 70% caprylic C_8_, 28% capric C_10_) Coconut based, solubilizer for poorly water soluble drugs	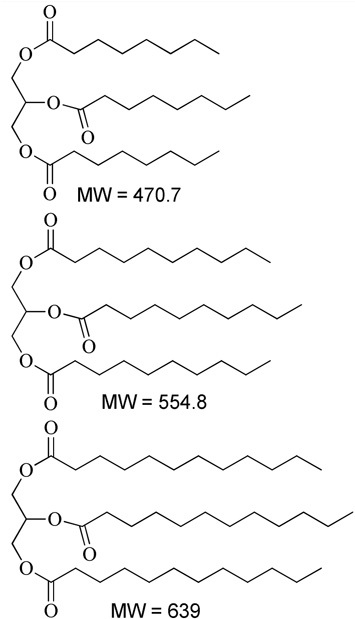	Cremer Oleo GmbH & Co. KG. Hamburg, Germany.
Kollisolv-MCT-70 (C6, C8, C10)	Primary oil (used as alternative excipient), Caprylic/capric acid triglycerides (2% caproic C_6_, 68% caprylic C_8_, 30% capric C_10_) Solubilizer for poorly water soluble drugs.	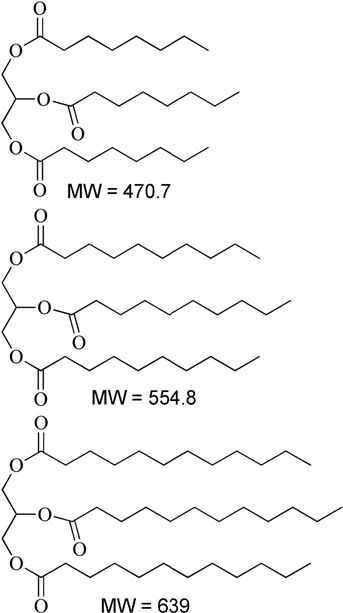	BASF CO. Germany

*Excipients can also be categorized based on their source, functional group, or their role in the formulation.*

### Experimental Methods

#### Fenofibrate SNEDDS Formulation Design

A number of similar lipid oils from various manufacturers (Table 1) with known surfactants were mixed to develop lipid-based formulations of (group a – SNEDDS) and (group b – SNEDDS). The formulations were prepared with varying concentrations of single or blend of two oils, and surfactant (% weight) by a simple two steps preparation method (Table 2). In the primary step, oil mixture was prepared with oils of different kinds from various manufacturers. Details of the chemical structures and origin of the materials are listed in Table 1. Then a surfactant was added to the primary mixture of oils at various ratios. The final mixture was vortexed to achieve homogeneity. The anhydrous formulation mix was kept in an airtight 3 mL glass vial prior to use. Afterward, the most attractive SNEDDS formulations (either group “a” or group “b”), which belong to LFCS Type III systems were investigated carefully using fenofibrate.

**Table 2:** Lipid formulations classification systems (LFCS) utilized and the % (w/w) composition of excipients in each formulation in this study.

LFCS type	No.	Formulation	Oil (%w)				Surfactant (%w)	
			C70	K70	CMCM	I742	TO106V	HCO30
I	F1a	C70	100	–	–	–	–	–
	F2b	K70	–	100	–	–	–	–
IIIA	F2a	C70:CMCM(7:3)/TO106V(1/1)	35	–	15	–	50	–
	F2b	K70:I742(7:3)/TO106V(1/1)	–	35	–	15	50	–
	F3a	C70:CMCM(7:3)/HCO-30(1/1)	35	–	15	–	–	50
	F3b	K70:I742(7:3)/HCO-30(1/1)	–	35	–	15	–	50
IIIB	F4a	CMCM/HCO30 (1/1)	–	–	50	–	–	50
	F4b	I742/ HCO-30 (1/1)	–	–	–	50	–	50

#### Visual Assessment and Determination of Formulation Clarity

Within the context of self-emulsifying efficiency, it is reported that a visual assessment is commonly accepted as initial determination of formulation efficiency. In the current study, self-emulsification properties of the formulation were assessed visually (Craig et al., 1995; Kommuru et al., 2001). Sample preparation was employed as follows: a 100 μL of each formulation was diluted with 50 mL of water in 50 mL volumetric flask (1:500 dilution) and agitated gently for 1 min. The miscibility, homogeneity and the appearance of dispersant were evaluated accordingly at room temperature.

#### Droplet Size Measurement, Polydispersity Index (PDI) and Zeta Potential

The droplet size of the self-emulsifying formulations and the use of excipients in formulation is a crucial factor to its performance by which the rate and extent of drug release as well as absorption can be estimated (Gursoy and Benita, 2004). The droplet size, PDI and zeta potential of the diluted LFCS Types I, III, and IV SEDDS/SNEDDS were measured by laser diffraction analysis using Zetasizer particle sizing systems (Model ZEN3600, Malvern Zetasizer nano series, United Kingdom). The formulations were diluted freshly at a ratio of 1:1000 w/v (SEDDS/SNEDDS: distilled water) and mixed for 1 min before analysis. The cuvette was used to fill diluted samples in the sample compartment and the data were collected for 10 times. All experiments were carried out in triplicates and represented as mean ± standard deviation.

#### Fenofibrate Loading Into SNEDDS

The fenofibrate loading into SNEDDS was determined using a shake flask solubility method to find a preconcentrate with maximum amount of drug (Shahba et al., 2012). The samples were prepared by adding an excess amount of drug to the formulation, which was then shaken and mixed thoroughly with a vortex mixer. The samples were incubated in a dry heat incubator at 37°C. After 7 days, samples were centrifuged in 1.5 mL Eppendorf at 13000 *g* to separate undissolved drug. An aliquot of the supernatant was weighed and diluted in an appropriate solvent to be analyzed using Ultra-High Performance Liquid Chromatography (UHPLC) method developed and published by our group (Mohsin et al., 2009). Three to six replicates were performed for each formulation. Data were presented as mean ± standard deviation.

#### Dynamic *in vitro* Dispersion of SNEDDS

Fenofibrate was dissolved in the representative SNEDDS at 80% saturation level based on its maximum drug loading attained by equilibrium solubility studies in the relevant anhydrous formulation. The formulations of SNEDDS, which were optimized by the characterization studies earlier, were included in the corresponding dynamic dispersion studies to investigate whether the drug will precipitate during dispersion in the intestinal aqueous media. The representative formulation (1 g) was dropped into 100 mL of aqueous media (milliQ water, pH-7.0, Fasted State Simulated Intestinal Fluid, pH-7.5- FaSSIF and Fed State Simulated Intestinal Fluid, pH-5.0- FeSSIF) and kept in a dry heat incubator at 37°C for 24 h. During this 24-h period, 1 mL of the dispersed sample from each vessel was withdrawn periodically (from 0 to 24 h), and centrifuged at 13000 *g*. A 100-μL aliquot of the resulting clear supernatant was assayed in triplicates by the UHPLC method (Alamri et al., 2017) to find out the amount of the drug remains dissolved in the samples.

#### Dynamic *in vitro* Lipolysis of SNEDDS Formulations: Preparation of Digestion Medium

For best clinical relevance, it is important to conduct *in vitro* drug tests to mimic the *in vivo* conditions as closely as possible. Earlier, the equilibrium solubility and dispersion characteristics of fenofibrate were determined using water and simulated intestinal aqueous media, which can be assumed to be representative of the conditions in the GI tract. *In vitro* lipolysis experiment is useful for two particular reasons. Firstly, to quantify the rate and extent of lipolysis by pH-stat titration process, which can establish how the formulation can be affected by these parameters. Secondly, after the reaction is terminated, the post lipolysis product can be analyzed to predict the fate of the drug after digestion and identify whether the drug is in solubilized form or precipitated out of intestinal contents. These models can reliably predict the ability of these formulations to enhance oral bioavailability of poorly water-soluble drugs.

In this study, the dynamic *in vitro* lipolysis experiment was a modified version of the procedure previously described by Mohsin (2012). For each digestion sample preparation, 250 mg of SNEDDS were dispersed into 9 mL of a simulated aqueous media (phospholipid/bile salt mixed micellar solution in digestion buffer made with SIF powder under fed and fasted conditions). The fed (FeSSIF) and fasted (FaSSIF) intestinal contents were simulated using 20 and 5 mM bile salt, respectively. Bile salts concentrations were 4 times higher than phospholipids concentrations in the digestion mixture (bile salt: phospholipid molar ratio 4:1), which is the ratio assumed to be secreted in bile. The SNEDDS formulations were emulsified by stirring continuously for 5–10 min in the mixed micellar solutions in a thermostatic jacketed glass reaction vessel at 37°C, prior to enzyme addition. Lipolysis of SNEDDS was initiated by addition of 1 mL pancreatic extract, containing 800 tributyrin units of pancreatic lipase/co-lipase. Lipolysis was continued for 30 min using a pH-stat titration unit (Metrohm, Switzerland), which was maintained a constant pH of 6.8. The fatty acids generated upon lipolysis were titrated with 0.2 M NaOH. The progress of lipid digestion in terms of lipolysis was monitored indirectly by pH-stat and directly by UHPLC analysis.

#### Determination of Fenofibrate Solubility in Simulated Intestinal Media During Lipolysis and in Post-lipolysis Products

To examine the solubility of the fenofibrate during the 30 min reaction time of lipolysis experiments, samples of the reaction medium were withdrawn immediately following the addition of lipase/co-lipase enzymes at serial time points subsequent to the initiation of lipolysis. 100 μL solution was collected for each assay at time 0 (just before enzyme addition), and then 1, 5, 10, and 30 min. Samples were then dissolved in methanol for UHPLC analysis.

At the completion of each digestion experiment two samples of 4.2 mL of digestion mixture were transferred into polyallomer centrifuge tubes (11 × 60 mm, Lot P60217, Beckman, Brea, CA, United States) and 40 μL of 4-bromophenyl-boronic acid was added to each sample to stop further lipolysis. Samples were then ultracentrifuged (model Optima XL-100K; Beckman, Palo Alta, CA, United States) at 334000 *g* for 30 min at 37°C. After ultracentrifugation, the SNEDDS digests were separated into an aqueous phase and a precipitated pellet phase. Samples obtained from each separated phases were analyzed for drug content by UHPLC. Each fraction was collected very carefully from each centrifuge tube in the following manner: The aqueous phase was aspirated into a 5-mL syringe by penetrating the side of the tube at least 0.5 cm above the pellet phase (without disturbing the pellet) with a 23-guage needle and transferred into 12-mL polypropylene tubes. Lastly, the pellet fraction was dissolved in chloroform: methanol (2:1 v/v) solution and transferred to a 5 mL volumetric flask. The pellet fractions were acidified with 100 μL of 1 M HCl and made up to volume with chloroform: methanol (2:1 v/v) solution. The digestion samples dissolved in chloroform: methanol (2:1 v/v) were sonicated for approximately 15 min at room temperature to make sure no insoluble particles remained. All the digestion samples were subsequently diluted 1 in 5 with methanol (v/v). Further suitable dilutions of the solution were also made in methanol (v/v) and analyzed by previously validated UHPLC techniques. Drug fractionation after ultracentrifugation of the digestion products is thought to indicate the likely fate of the drug after lipolysis (Pouton, 2000). Drug molecules solubilized in the aqueous phase of the lipolysis medium are assumed to be available for absorption. Therefore, the aqueous phase was of greatest interest in the study of the GI absorption of fenofibrate.

#### Analysis of the Fenofibrate in SNEDDS

The quantity of fenofibrate in SNEDDS samples was determined by a highly sensitive UHPLC system that consisted of a Dionex^®^ UHPLC binary solvent manager equipped with a Dionex^®^ automatic sample manager and a Photodiode Array (PDA) detector (Thermo scientific, Bedford, MA, United States). An Acquity^®^ UPLC BEH C_18_ column (2.1 × 50 mm, 1.7 μm); kept at 25°C was used for the drug analysis. The mobile phase was an isocratic mixer of methanol and water in a ratio of 65:35% (v/v). Freshly prepared mobile phase was filtered through an online 0.20 μm filter and degassed continuously by an online degasser within the UHPLC system. The flow rate of the mobile phase was 0.3 mL/min with total run time of 2.5 min. The detector wavelength was set at 284 nm and the injection volume was 1.0 μL. The developed method was validated as per US Department of Health and Human Services, Food Drug Administration guidelines (US Department of Health and Human Services Food and Drug Administration et al., 2001). The linearity of the method was found to be suitable in the range of 0.1–10 μg/mL (*r*^2^ = 0.9993).

#### *Ex vivo* Permeation Study

The intestinal permeation of ideal formulation (F3b) was compared with the raw fenofibrate dispersion using non-everted sac technique (Ibrahim et al., 2014). Healthy male wistar rats, weighing 200–250 g, were used. All studies were in accordance with the Guidelines of Animal Ethical Committee of King Saud University. Animals were reserved without food overnight but water was permitted ad libitum before experiment. Animals were sacrificed under ether anesthesia and jejunum was quickly cut into segments, each about 5–7 cm in length. The segments were washed immediately in oxygenated Krebs solution (pH 6.5). The Krebs was composed of 7 g/L sodium chloride, 0.34 g/L potassium chloride, 1.8 g/L glucose, 0.251 g/L disodium hydrogen phosphate, 0.207 g/L sodium dihydrogen phosphate, and 46.8 mg/L magnesium chloride (Alam et al., 2012). Sac of each segment was tied on one side with a silk suture and filled with raw fenofibrate suspension (1 mL, 100 μg/mL) or F3b formulation (equivalent to 100 μg/mL in 1 mL). Then, the sacs were tied on the other side and incubated into pre-warmed (37°C) and pre-oxygenated Krebs solution in a 10 mL tubes. Aliquots of 0.5 mL were withdrawn at certain time intervals as 15, 30, 45, 60, 75, 90 and 120 min and replaced with an equal fresh pre-heated aliquot. The amount of fenofibrate transported through the intestinal segments was determined using UHPLC. The apparent permeability was then calculated based on the following equation (Mahmoud and Ahmed, 2004):

$$\text{Papp}=\frac{\text{dQ}}{\text{dt}}\times \frac{1}{\text{CoA}}$$

Where dQ/dt is the rate of drug permeation, C_0_ is the initial concentration of the donor and A is the surface area of intestine (cm^2^).

#### Statistical Analysis

Prism pad^®^ (4.5 version for Windows, GraphPad Software, Inc., La Jolla, CA, United States) was utilized to explore the significance of the data. Droplet size data were compared using Paired *T*-test. The one-way ANOVA test followed by *post hoc* analysis was employed to compare the *in vitro* dispersion, digestion and permeability profiles. A *P-*value equal and or less than 0.05 was considered statistically ‘significant.’

## Results and Discussion

### Visual Assessment and Clarity of the Formulation

Visual observation is a plausible mean of assessment to the experienced eye in order to differentiate efficient and poor formulations since most of these formulations are expected to produce nano-sized droplets upon dispersion. The following factors were considered for visual assessments to optimize SNEDDS formulation: homogeneity, time for dispersion and appearance upon dilution with water. The dilution ratio of 1:500 (formulation: water) was maintained (see Figure 1). In the current study, efficient formulations are defined when homogeneous dispersion (transparent/bluish appearance in Figure 1) is achieved in less than 1 min. As shown in Table 3, all representative formulations except F1a and F1b (non-dispersible in aqueous media) were found to be efficient and were used for further optimization.

**Table 3:** Visual assessment data of dispersions formed by various formulation systems (“group a” and “group b”) under conditions of self-emulsification.

LFCS type	No.	Formulation	HOMO	DISP	APPE	SNEDDS
I	F1a	C70	No	N/A	Turbid	×
	F1b	K70	No	N/A	Turbid	×
IIIA	F2a	C70:CMCM(7:3)/TO106V(1/1)	Yes	∼5 s	Hazy	√
	F2b	K70:I742(7:3)/TO106V(1/1)	Yes	<1 min	Transparent	√
	F3a	C70:CMCM(7:3)/HCO-30(1/1)	Yes	∼5 s	Hazy	√
	F3b	K70:I742(7:3)/HCO-30(1/1)	Yes	<1 min	Transparent	√
IIIB	F4a	CMCM/HCO30 (1/1)	Yes	<1 min	Bluish	√
	F4b	I742/HCO-30 (1/1)	Yes	<1 min	Transparent	√

*Values were cross checked for the determinations of each formulation. HOMO, homogeinity; DISP, dispersibility; ^∗^APPE, appearance.*

### Effect of Excipients on Formulation Characteristics

There are two criteria commonly proposed to define the efficiency of self-emulsifying formulation: (a) the emulsification rate and (b) the droplet size distribution of the produced emulsion. Our droplet size analysis, polydispersity index (PDI) and zeta potential results, shown in Table 4, clearly indicated that the self-emulsification efficiency is strongly associated with the mean droplet size of the resultant emulsion, which is consistent with previous reports (Charman et al., 1992; Humberstone and Charman, 1997).

**Table 4:** The particle size and zeta potential (mean ± SD, *n* = 3–6) of various LFCS formulations.

No.	Formulation	Particle size Z-Ave (nm)	PDI	Zeta potential (mV)
F1a	C7O	1066 ± 30	0.874	−0.916 ± 89.7
F1b	K7O	1022 ± 23	0.559	−4.78 ± 74.2
F2a	C70:CMCM(7:3)/TO106V(1/1)	213.81 ± 2.05	0.279	−30.4 ± 5.42
F2b	K7O:I742(7:3)/TO106V(1/1)	179.13 ± 6.75	0.232	−33.5 ± 7.58
F3a	C70:CMCM(7:3)/HCO-30(1/1)	59.48 ± 0.13	0.237	−19.9 ± 17.2
F3b	K7O:I742(7:3)/HCO-30(1/1)	45.74 ± 0.07	0.224	−31.5 ± 5.78
F4a	CMCM/HCO30(1/1)	64.38 ± 7.13	0.211	−22.6 ± 6.61
F4b	I742/HCO-30(1/1)	36.79 ± 0.14	0.165	−23.4 ± 5.75

*Formulations were dispersed in water at 1:1000 dilutions level (w/v).*

The PDI provides information on the homogeneity of the particle size distribution of the formulation by measuring the width of the particle dispersion. According to the report of the literature the PDI value of less than 0.3 are considered ideal and depicts a narrow size distribution (Pathak and Nagarsenker, 2009). Therefore, according to Table 4, all the formulations had a narrow size dispersion except formulations F1a and F1b, which have PDI index of 0.874 and 0.559, respectively.

All the prepared formulations showed a droplet diameter within 36.79–213.81 nm except for formulations F1a and F1b, which formed particles with an average size of 1066 and 1022 nm, respectively. Although formulation F2a showed hazy appearance upon dispersion, it was considered efficient SNEDDS as it produced 213.81 nm droplet sizes (Table 4). The rest of the formulations were used as SNEDDS due to their clarity of the dispersions (bluish/transparent) as well as droplet sizes below 200 nm.

The type of lipid excipients used in each group of formulations also had a significant (*P* < 0.05) effect on the zeta potential (−0.916 mV in F1a, −4.78 mV in F1b, and −19.9 mV in F3a, −31.5 mV in F3b). However, no statistically significant difference (P.0.05) was noticed between F4a (−22.6 mV) and F4b (−23.4 mV) which were the most hydrophilic formulations (Table 4). In addition, the zeta potential was increased for F3 formulations to the range of −30.4 mV (F3a) to −33.5 mV (F3b). Among all formulations, the lowest droplet sizes were recorded for F3b and F4b (36.79 nm and 45.74 nm, both formulations contained I742).

Changing the lipid excipients combination such as Cremercoor (C70)/Capmul MCM (CMCM) Vs Kollisolv (K70)/Imwitor 742 (I742) with the particular surfactant in all formulations have significant effects (*P* < 0.05) on particle size and zeta potential.

The overall droplet size and zeta potential data indicated the pivotal role of excipients. The formulations containing Kollisolv MCT and or Imwitor 742 yielded comparably lower size ranges with higher comparative zeta potential values than that of Cremercoor MCT and or Capmul MCM upon aqueous dispersion. Therefore, Kollisolv MCT and or Imwitor 742 are more appropriate to develop SNEDDS formulations with relatively low particle size and high stability.

Droplet size upon aqueous dispersion plays key role in oral absorption of the drug *in vivo*. The smaller the droplet size the larger the interfacial surface area will be provided for drug absorption (Gershanik and Benita, 2000; Pouton, 2000; Kang et al., 2004). Nevertheless, it should be recognized that the dispersion may be modified substantially by digestion *in vivo*.

Photon correlation spectroscopy (PCS) method is common and widely used for determination of emulsion droplet size (Wakerley and Pouton, 1987; Craig et al., 1995; Gershanik and Benita, 2000; Gursoy and Benita, 2004). The PCS technique can best fit if the emulsion properties of the formulations are not changed following the substantial aqueous dilutions necessary for applying this method. Based on the droplet size, SNEDDS can be easily distinguished by PCS, as less than 200 nm leads to formation of SNEDDS (nanocarriers), which are stable, isotropic and transparent aqueous dispersions (Vonderscher and Meinzer, 1994; Constantinides, 1995). However, particle size of the initial dispersion is not the only determinant of lipid-based delivery systems absorption as it is also governed by their fate in the gastrointestinal tract (GIT).

### Fenofibrate Loading and Effect of Excipients

**FIGURE 2:**
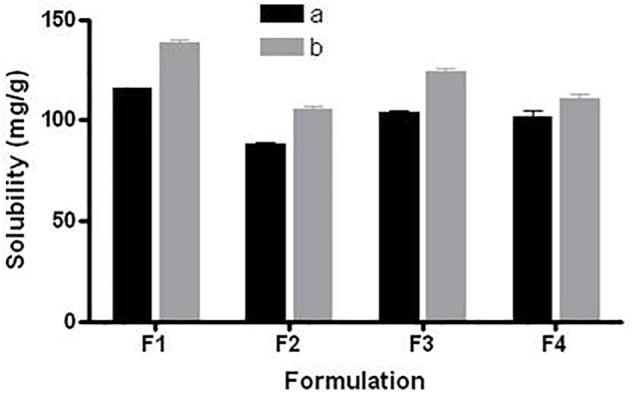
Equilibrium solubility of fenofibrate in group “a” and group “b” of LFCS formulations containing alternation lipid excipients. The formulation represent F1a- C70, F1b- K70, F2a- C70: CMCM (7:3)/TO106V (1/1), F2b- K70:I742(7:3)/TO106V(1/1), F3a- C70: CMCM (7:3)/HCO-30 (1/1), F3b- K70:I742(7:3)/HCO-30 (1/1), F4a- CMCM/HCO-30 (1/1) and F4b - I742/HCO-30 (1/1) respectively. Data are expressed as Mean ± SD, (*n* = 3).

Equilibrium solubility is an important part for developing any drug formulation because it provides the necessary information for loading maximum dose that can be incorporated in a single unit capsule/tablet. All anhydrous formulations were kept in this study for 7-days at 37°C temperature as a standard period to make sure that the equilibrium has been achieved. Equilibrium solubility data of fenofibrate in various (group a) and (group b) LFCS Types I, III and IV lipid-based formulations (8 formulations) is presented in Figure 2.

Among all the representative formulations (F1–F4), fenofibrate was significantly soluble in the SNEDDS formulations containing Kollisolv MCT and or I742 compared to Cremercoor MCT and or CMCM as excipients. Kollosolv MCT alone (F1b) was able to solubilize 138.44 mg/g fenofibrate, which was the highest amount compared to other formulations (Figure 2). Fenofibrate solubility was considerably higher in the most promising Type IIIA systems where it was between 88.37 mg/g to 124.07 mg/g, whereas F3b could solubilize 124.07 mg, which was the highest amount of drug. In formulation Type IIIB, although fenofibrate solubility was dropped slightly down to 101.55–110.32 mg/g, Imwitor 742 showed improved drug entrapment than Capmul MCM. The higher solubility in Type IIIA suggests that fenofibrate can be dissolved in higher amount in SNEDDS formulations containing mix glycerides (mix mono-, di- and triglycerides of group b, Kollisolv MCT/Imwitor 742) and surfactant of moderate HLB (HCO-30, HLB 11). From the overall solubility studies, it can be stated that fenofibrate is more suitable with Kollisolv MCT/Imwitor 742 blended formulations than their counterpart Cremercoor MCT/Capmul MCM blended formulations (Figure 2).

### *In vitro* Dynamic Dispersion and Drug Precipitation in Intestinal Media

Dynamic *in vitro* dispersion tests evaluate the ability of lipid-based vehicles to disperse into various types of media, and to assess whether the drug partitions from the vehicle into the aqueous medium or stays in solubilised form prior to absorption into systemic circulation. The justification for conducting this test to predict the *in vivo* performance is established upon the fact that the solubilized drug is readily available for absorption, while the precipitated drug is not. Hence, this test shows that the percentage of solubilized drug in biological fluids *in vitro* could be correlated with the rate and extent of drug absorption *in vivo*. Therefore, the dispersion tests, could guide drug development and selection of appropriate formulations for further *in vivo* studies. Dressman and colleagues have developed experimental methodologies in a range of biorelevant dissolution test media that have established application in drug release studies from lipid-based oral formulations (Dressman and Klein, 2005; Dressman et al., 2007).

A standard dissolution apparatus can be appropriate to conduct dispersion tests but, assuming that the drug in solution is initially in the anhydrous formulation, the emphasis should be on estimating unwanted precipitation of the drug that is likely to occur prior to digestion rather than dissolution/drug release. In this study, samples were withdrawn from the dispersion vessel at various intervals within 24 h and analyzed to determine the likelihood of precipitation during GI transit.

**FIGURE 3:**
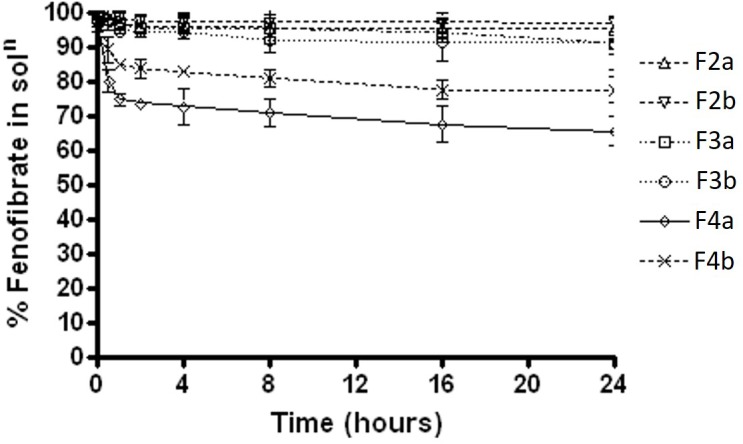
The % fenofibrate in solution during 24 h time after 1:100 dilution in the aqueous media (water) (fenofibrate was dissolved at 80% of the equilibrium solubility in the anhydrous mixture). The formulation represent F2a- C70: CM (7:3)/TO106V (1/1), F3a- C70: CM (7:3)/ HCO-30 (1/1), F2b- K70:I742(7:3)/TO106V(1/1), F3b- K70:I742(7:3)/HCO-30 (1/1), F4a- CMCM/HCO-30 (1/1) and F4b- I742/ HCO-30 (1/1) respectively. Data are presented as mean ± SD, (*n* = 3).

Fenofibrate precipitation experiments was firstly carried out in water medium over 24 h. The results showed that formulations F2a and F2b, which contain Type IIIA with water insoluble surfactant, maintained almost 98% drug as solubilised form in aqueous solution, whereas F3a and F3b, which contain Type IIIA with water soluble surfactant, maintained approximately 95%. On the other hand, the Type IIIB SNEDDS formulations 4a and 4b, which contained predominantly water soluble materials were able to maintained 65 and 82% fenofibrate in solution, respectively (Figure 3).

**FIGURE 4:**
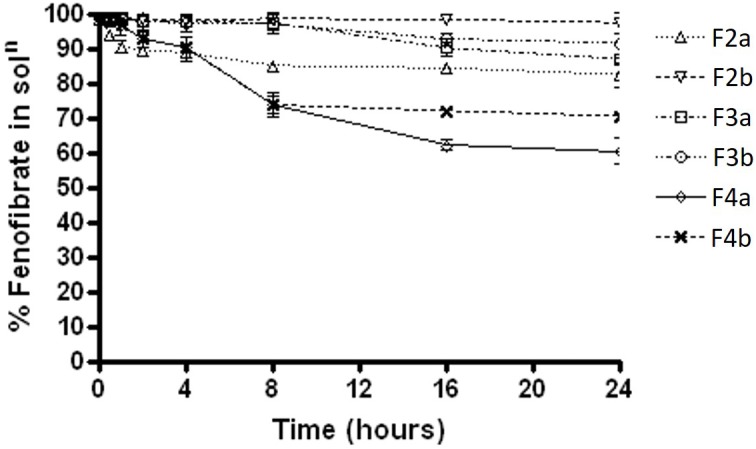
The % fenofibrate in solution during 24 h time after 1:100 dilution in the simulated fasted state intestinal media (fenofibrate was dissolved at 80% of the equilibrium solubility in the anhydrous mixture). The formulation represent F2a- C70: CM (7:3)/TO106V (1/1), F3a- C70: CM (7:3)/ HCO-30 (1/1), F2b- K70:I742(7:3)/TO106V(1/1), F3b- K70:I742(7:3)/ HCO-30 (1/1), F4a- CMCM/HCO-30 (1/1) and F4b- I742/ HCO-30 (1/1) respectively. Data are presented as mean ± SD, (*n* = 3).

The results were quite different when the dispersion experiment was conducted in FaSSIF medium. Substantial precipitations were occurred in all the formulations except F2a and F2b. Only, F2a and F2b SNEDDS showed greater performance in maintaining fenofibrate in solution (approximately 98%). However, F3a had more precipitation rate than F3b (35% drug precipitated) and in a similar way F4a (80% drug precipitated within 16 hrs of time) had significant precipitation rate than F4b (38% drug precipitated after 24 h) (Figure 4).

**FIGURE 5:**
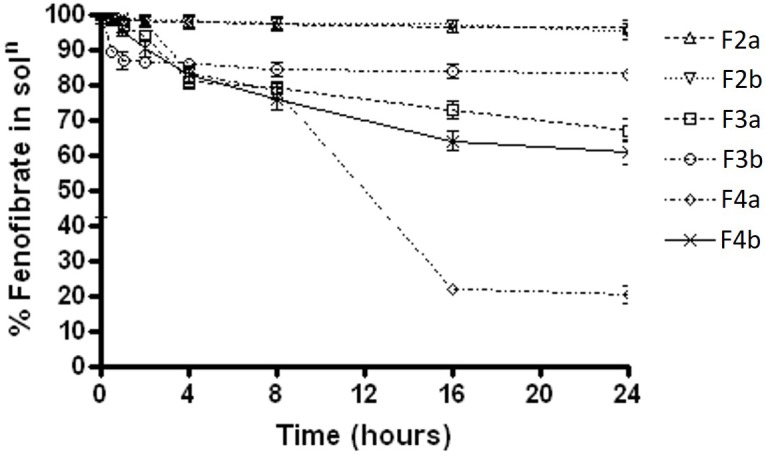
The % fenofibrate in solution during 24 h time after 1:100 dilution in the simulated fed state intestinal media (fenofibrate was dissolved at 80% of the equilibrium solubility in the anhydrous mixture). The formulation represent F2a- C70: CM (7:3)/TO106V (1/1), F3a- C70: CM (7:3)/ HCO-30 (1/1), F2b- K70:I742(7:3)/TO106V(1/1), F3b- K70:I742(7:3)/HCO-30 (1/1), F4a- CMCM/HCO-30 (1/1), and F4b- I742/HCO-30 (1/1) respectively. Data are presented as mean ± SD, (*n* = 3).

In FeSSIF media, F2b, F3a, and F3b maintained almost 99% of the drug for 8 h time (Figure 5), then started precipitating out gradually. However, only F2b within group b – SNEDDS kept most of the drug (>99%) concentration in solubilised form until 24 h. On the other hand, all other formulations kept 90% drug in solution unto 8 h and then precipitated substantially. The data in Figure 5 showed that F4a was the worse formulation as precipitated more than 35% drug out of solution. The overall dispersion study confirmed that blended polar mixed glycerides of Kollisolv MCT/Imwitor 742 (group b) were able to retain high percent drugs in solution for more than 24 hrs time in all aqueous media. Thus the bioavailability of fenofibrate can be significantly increase if it stays in solubilised form, during the digestion time (around 4 h) *in vivo* with the representative group b – SNEDDS systems.

The proposed study in this research shows that, despite the diversity and complexity, there are general considerations in digestion of lipids that one can follow in selecting and developing SNEDDS formulation of different groups for poorly water-soluble drugs.

To maintain drug in solution by avoiding precipitation upon dispersion is the desired goal for successful application of pharmaceutical products. Correlations between the investigations of the equilibrium solubility of the drug in the aqueous diluted formulation, and corresponding dynamic precipitation tests could help to predict whether precipitation is likely to take place, and whether it would affect bioavailability (Mohsin et al., 2009). Increasing the solubilization capacity of the formulation extensively over the desired drug concentration could help avoid *in vivo* drug precipitation by minimizing drug loading in unit dose. Another possible method, which is commonly used at present to slow down or prevent drug precipitation, is utilizing hydrophilic polymeric ingredients in the formulation that act as precipitation inhibitors (Gao et al., 2003; Gao et al., 2004; Gao and Morozowich, 2007).

### Assessment of Post Digestion Products After *in vitro* Dynamic Lipolysis

One of the most complex and poorly understood aspects of lipid-based oral formulations is their interaction with the GI lipid digestion system and the influence that these interactions have on their performance. Digestion of dietary triglyceride in the small intestine is usually very rapid, and several other non-ionic esters, such as mixed glycerides and surfactants, will be substrates of pancreatic lipase or other esterases. Lipid digestion of the formulation may facilitate the dispersion of the drug as SNEDDS in presence of bile salt/phospholipids and promotes its absorption. Therefore, lipid digestion experiments can be of great importance because they offer the opportunity to predict the fate of the formulation and drug in the intestinal lumen.

Within the scope of the present studies, changes in solubilization capacity that occurred during digestion of formulation components was of great importance to assess in detail using *in vitro* lipolysis. The *in vitro* lipolysis experiments under fed and fasted conditions were as true a model of the intestine as one can expect to achieve maximum *in vitro.* Taken together these two tests provide an understanding of what can be expected *in vivo*. Although Type IIIA formulations, based on medium-chain excipients, can maintain fenofibrate in solution after dispersion, the drug is likely to precipitate upon digestion. Data obtained in this study facilitates further deep investigation to have a database on lipid digestion and the solubilization of a range of lipid formulations, and thus assists in the more rational design of new lipid-based formulations. The present study proved that representative SNEDDS was digested to completion very quickly and is not dependent on bile. Medium-chain mixed glycerides are good solvents for fenofibrate as readily digested and would be expected to be completely digested *in vivo* to FAs and glycerol.

#### Percent Fenofibrate Available in Solution During Lipolysis Reaction in Presence of Lipase

**FIGURE 6:**
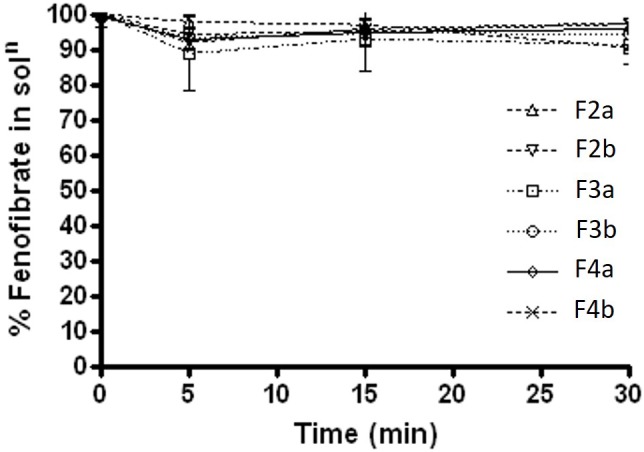
Fenofibrate concentration (mg/mL) in solution during 30 min lipolysis experiments under fasted condition. The formulation represent F2a- C70: CM (7:3)/TO106V (1/1), F3a- C70: CM (7:3)/HCO-30 (1/1), F2b- K70:I742(7:3)/TO106V(1/1), F3b- K70:I742(7:3)/HCO-30 (1/1), F4a- CMCM/HCO-30 (1/1), and F4b- I742/ HCO-30 (1/1) respectively. Data are presented as mean ± SD, (*n* = 3).

**FIGURE 7:**
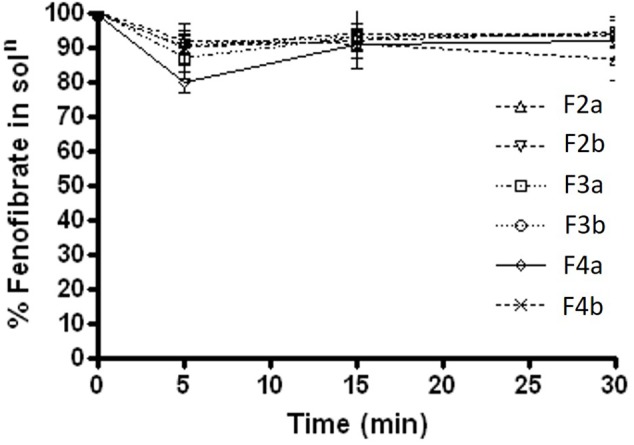
Fenofibrate concentration (mg/mL) in solution during 30 min lipolysis experiments under fed condition. The formulation represent F2a- C70: CM (7:3)/TO106V (1/1), F3a- C70: CM (7:3)/ HCO-30 (1/1), F2b- K70:I742(7:3)/TO106V(1/1), F3b- K70:I742(7:3)/ HCO-30 (1/1), F4a- CMCM/HCO-30 (1/1), and F4b- I742/ HCO-30 (1/1) respectively. Data are presented as mean ± SD, (*n* = 3).

During the lipolysis experiments, it was crucial to investigate if there was any drug loss or precipitation occurred within 30 min of experimental time under fed and fasted conditions. The results under fasted state (Figure 6) showed that fenofibrate was in solubilised form with most formulations except for Type IIIB (F4a and F4b, contained water soluble materials predominantly), which lead to approximately 3–7% precipitations. The results were quite similar under fed state shown in Figure 7, where the drug loss was estimated to be as low as 3–7% after 30 min of formulation digestion. In both fed and fasted conditions, the results suggested that Type IIIA and IIIB SNEDDS were able to keep fenofibrate in solubilised state in presence of lipase within 30 min time, which is crucial for lipid digestion.

#### Solubilization of Drugs in Digestion Products and %Total Recovery

The distribution and solubilization pattern of fenofibrate across the different phases of the digestion medium are summarized in Table 5. The data shows the percent of fenofibrate found in aqueous phase (AP) and pellet phase (PP) under fed and fasted conditions after a 30 min digestion period.

**Table 5:** % fenofibrate in aqueous phase (AP) and pellet phase (PP) after 30 min digestion under fed and fasted condition, (*n* = 3 ± SD).

No.	Formulation	Fasted			Fed		
		% Drug in AP	% Drug in PP	%Tot. Rec.	% Drug in AP	% Drug in PP	%Tot. Rec.
F2a	C70:CMCM(7:3)/TO106V (1/1)	9.22 ± 0.67	79.23 ± 1.23	88.45	8.99 ± 1.11	78.99 ± 0.77	87.98
F2b	K70:I742(7:3)/To-106V(1/1)	11.23 ± 3.23	84.79 ± 0.87	96.02	10.09 ± 0.21	83.36 ± 1.07	93.45
F3a	C70:CMCM(7:3)/ HCO-30 (1/1)	08.24 ± 0.00	78.72 ± 0.20	86.96	07.20 ± 0.61	85.44 ± 0.02	92.64
F3b	K70:I742(7:3)/HCO-30 (1/1)	10.18 ± 0.00	83.41 ± 0.80	93.59	4.84 ± 0.00	88.68 ± 1.11	93.52
F4a	CMCM/HCO-30 (1/1)	7.64 ± 2.11	84.81 ± 0.44	92.45	7.43 ± 1.32	84.23 ± 3.11	91.66
F4b	I742/ HCO-30 (1/1)	9.79 ± 0.03	86.75 ± 0.00	96.54	8.75 ± 0.50	85.29 ± 0.93	92.04

*Concentration of fenofibrate (mg) in mixed BS micelles before and after digestion under fed conditions. The data represent the original mass of fenofibrate which was recovered from the aqueous and pellet phases after digestion.*

The data from digestion studies demonstrate that formulation development using medium-chain mixed glycerides leads to extremely low amounts of solubilized drug in the AP around 6–12% of the drug dose. However, the remaining large quantity of the drug was found in the PP around 84–90% Table 5). There was a small quantity of drug was also missing from most of the SNEDDS systems during the 30 min digestion time, which was not detected as fenofibrate during the UHPLC analysis. Nevertheless, all the formulations maintain less amount of drug in AP but Kollisolv MCT contained SNEDDS had higher solubilised fenofibrate in AP than the SNEDDS contained Cremercoor MCT and also total drug recovery was improved (Table 5). However, the mixed glycerides oil was not completely digested within 30 min so it is unclear to us to what extent solubilization could be maintained after complete digestion. Drug molecules that precipitated during lipolysis in the intestine are not expected to be available for absorption, at least not until slow re-dissolution step has been accomplished. The drug presented in the pellet phase may be in solubilized form to be available in the intestine for absorption.

The extent of solubilization of fenofibrate in the aqueous phase after digestion was slightly increased (P, 0.05) under fasted conditions, compared to fed conditions, indicating that the capacity of bile-lipid mixed micelles is likely to be a limiting factor. The total mass of drug recovered in the lipolytic products of both aqueous and pellet phase after completion of the lipolysis was often less than the original dose dissolved in the formulations. This may have been explained by hydrolysis of fenofibrate to fenofibric acid during lipolysis. The inclusion of surfactant TO106V and HCO-30, which combined well with medium chain mono-, di- and triglycerides (Cremercoor MCT) representing F2a, F3a SNEDDS systems produced relatively hazy/coarse emulsions on dilution with BS/PL solutions, whereas with Kollisolv MCT, F2b and F3b produced fine/transparent emulsions on dilution with BS/PL solution. The digestions of F2a andF3a formulations resulted in a lower mass of drug in the AP than F2b and F3b formulations, respectively. The Type IIIB formulations of F4a and F4b systems had low fenofibrate solvent capacity upon dispersion and digestion under physiological media. This was confirmed by the experimental assessment during digestion where the majority of the drug was assumed to be in the pellet phase as precipitates after digestion.

Since the recovery of fenofibrate from the digestion experiments appeared to be consistently lower than the original mass used, it was of interest to perform a mass balance test before, during the early stages and after *in vitro* digestion. Table 5 represent the total recovery of fenofibrate in the digestion products (both aqueous and pellet phase) of the formulations before addition of pancreatin, and after 1, 5, 15, and 30 min of digestion in the presence of pancreatin.

Fenofibrate was apparently missing after digestion of most of the SNEDDS formulations. The data suggest that mass balance is highly needed prior to addition of pancreatin but that there was a consistent shortfall in recovery once digestion commences, and the shortfall appeared to be consistently greater after 30 min. However, if the shortfall is due to hydrolysis of fenofibrate then the hydrolysis must be affected markedly by formulation. Given that it was not possible to identify a clear UHPLC peak for fenofibric acid, the most likely explanation was that fenofibrate increasingly becomes sequestered in the complex multiphasic mixtures resulting from digestion by pancreatin.

#### *Ex vivo* Permeation Study

**FIGURE 8:**
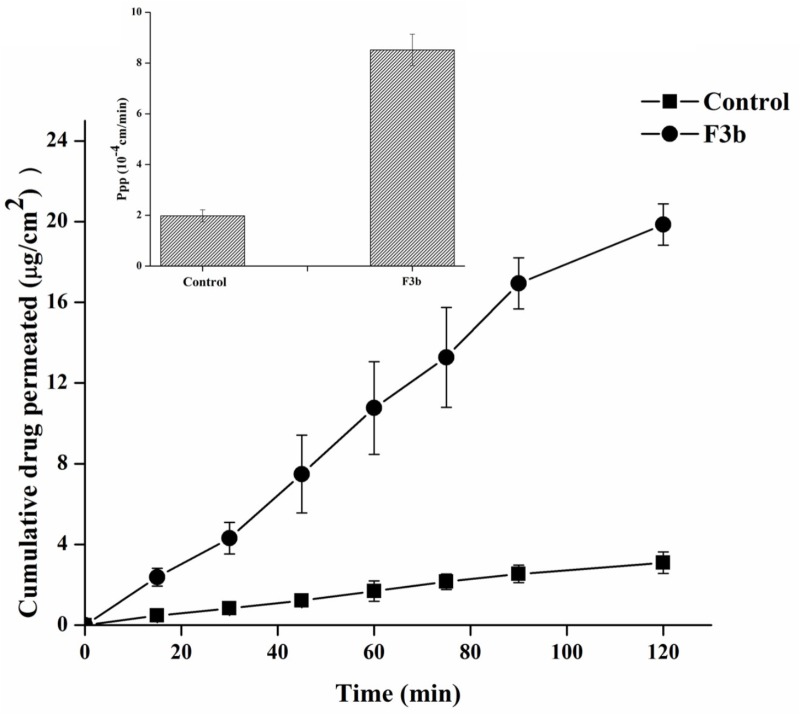
*Ex vivo* permeation profiles and Papp of fenofibrate from raw dispersion and SNEDDS model formulation F3b using non-everted rat intestinal sac model.

The permeation of fenofibarte from F3b SNEDDS versus their corresponding pure drug as dispersion was assessed using non-everted “intestinal sac technique” as shown in Figure 8. Ruan et al. (2006) revealed a consistency between the permeation by non-everted rat intestinal sacs technique of 11 marketed compounds model and their human absorption data. Therefore, it was used to predict *in vivo* absorption of the drugs. It was observed that the permeability of fenofibrate dispersion was slowly increased with time from 0.474 ± 0.175 μg/cm^2^ until it reached a value of 3.094 ± 0.135 μg/cm^2^ after 2 h. However, the permeability of F3b, fenofibrate was significantly improved compared with the control, reaching values of 19.855 ± 5.142 μg/cm^2^ after 2 h. Furthermore, Papp for F3b was found to be 8.518 × 10^−4^ cm/s (*p* < 0.005), which was 4.3 fold as compared to raw fenofibrate dispersion (1.982 × 10^−4^ cm/s). This means that the SNEDDS formulation caused an enhancement of the membrane permeation of fenofibrate. This could be attributed to the noticeable reduction of particle size and enhancement of surface area, which lead to a high rate of drug dissolution and diffusion. Likewise, SNEDDS could be ascribed to high concentration of fenofibrate due its solubility in the emulsion and promising partitioning of the drug (Kim et al., 2005). Moreover, the presence of the drug in the internal part of emulsification system may shield it from gut enzymes and avoid its degradation. In addition, SNEDDS can permeate the mucous layer and release the drug directly on the surface of the cell membrane.

## Conclusion

The SNEDDS containing Kollisolv MCT have shown high potential to improve the solubility and maintain fenofibrate in solubilised state prior absorption. The selection of suitable excipients, such as oils, and surfactants for fenofibrate is considered as a critical step to design a SNEDDS and their further performance. The selection of the most appropriate lipid excipients in formulation can only be made after undertaking *in vitro* dispersion studies and assessing the likely performance of the formulation *in vivo*.

Consequently, it was revealed that the choice of core lipid has a much greater impact on the solubilisation than the postprandial state during digestion. The present investigation suggests that SNEDDS can significantly enhance solubilisation of poorly water-soluble lipophilic drug, fenofibrate during digestion and *ex vivo* permeation which potentially could provide manufacturing and storage advantages while maintaining or possibly even enhancing bioavailability compared to regular liquid SNEDDS formulations.

## Author Contributions

AA performed the formal experiments in the study and prepared the original draft of the manuscript. MK provided the technical support and analyzed the data and obtained the funding. MB conducted the intestinal permeability experiments and revised the entire manuscript. FA contributed to editing and critically revising the manuscript for important intellectual content. All coauthors approved the final version of the manuscript for submission.

## Conflict of Interest Statement

The authors declare that the research was conducted in the absence of any commercial or financial relationships that could be construed as a potential conflict of interest.
